# A Review of Avian Influenza A Virus Associations in Synanthropic Birds

**DOI:** 10.3390/v12111209

**Published:** 2020-10-23

**Authors:** Susan A. Shriner, J. Jeffrey Root

**Affiliations:** National Wildlife Research Center, US Department of Agriculture, Animal and Plant Health Inspection Service, Wildlife Services, Fort Collins, CO 80521, USA; jeff.root@usda.gov

**Keywords:** avian, crow, European starling, house sparrow, influenza A virus, peridomestic, passerine, pigeon, synanthropic, wildlife–agriculture interface

## Abstract

Avian influenza A viruses (IAV) have received significant attention due to the threat they pose to human, livestock, and wildlife health. In this review, we focus on what is known about IAV dynamics in less common avian species that may play a role in trafficking IAVs to poultry operations. Specifically, we focus on synanthropic bird species. Synanthropic species, otherwise known as peridomestic, are species that are ecologically associated with humans and anthropogenically modified landscapes, such as agricultural and urban areas. Aquatic birds such as waterfowl and shorebirds are the species most commonly associated with avian IAVs, and are generally considered the reservoir or maintenance hosts in the natural ecology of these viruses. Waterfowl and shorebirds are occasionally associated with poultry facilities, but are uncommon or absent in many areas, especially large commercial operations. In these cases, spillover hosts that share resources with both maintenance hosts and target hosts such as poultry may play an important role in introducing wild bird viruses onto farms. Consequently, our focus here is on what is known about IAV dynamics in synanthropic hosts that are commonly found on both farms and in nearby habitats, such as fields, lakes, wetlands, or riparian areas occupied by waterfowl or shorebirds.

## 1. Introduction

Avian influenza A viruses (IAVs) have received significant research attention due to the threat these viruses pose to human, livestock, and wildlife health. Wild birds common at the wildlife–agricultural interface have a high potential to share viruses with poultry when spillover occurs from wildlife to poultry, and then potentially spills back from poultry to wildlife [1]. While most naturally occurring wild bird IAVs cause mild or no clinical signs in their wild bird hosts, these viruses exhibit extensive subtypic diversity based on surface proteins. All 16 hemagglutinin, or HA, subtypes have been isolated in wild birds along with nine different neuraminidases [2,3,4,5]. When wild bird IAVs spill over into poultry they are generally low pathogenic (LP) to chickens [6]. However, when H5 and H7 IAV subtypes spill over into poultry they have the potential to mutate and adapt, and can emerge as highly pathogenic (HP) strains that can cause significant health and economic harm to humans, poultry, and wildlife [6,7,8]. Therefore, understanding how wild bird IAVs spill over and spread in poultry is a high priority for controlling the emergence of high consequence IAV strains.

In this review, we focus on what is known about IAV dynamics in less common avian hosts. Specifically, we focus on synanthropic bird species. Synanthropic species, also known as peridomestic species, are species that are ecologically associated with humans and anthropogenically modified landscapes, such as agricultural and urban areas. Aquatic birds, such as waterfowl and shorebirds, are the species most commonly associated with IAVs, and are generally considered reservoir or maintenance hosts in the natural ecology of these viruses [2]. Waterfowl and shorebirds are occasionally associated with poultry facilities, but are uncommon or absent in many areas, especially on large commercial facilities. In these cases, spillover hosts that share resources with both maintenance hosts and target hosts such as poultry may play an important role in introducing wild bird viruses onto farms. Consequently, our focus in this review is on what is known about IAV in synanthropic hosts that are commonly found on both farms and in nearby habitats, such as fields, lakes, wetlands, or riparian areas occupied by waterfowl or shorebirds.

**Figure 1:**
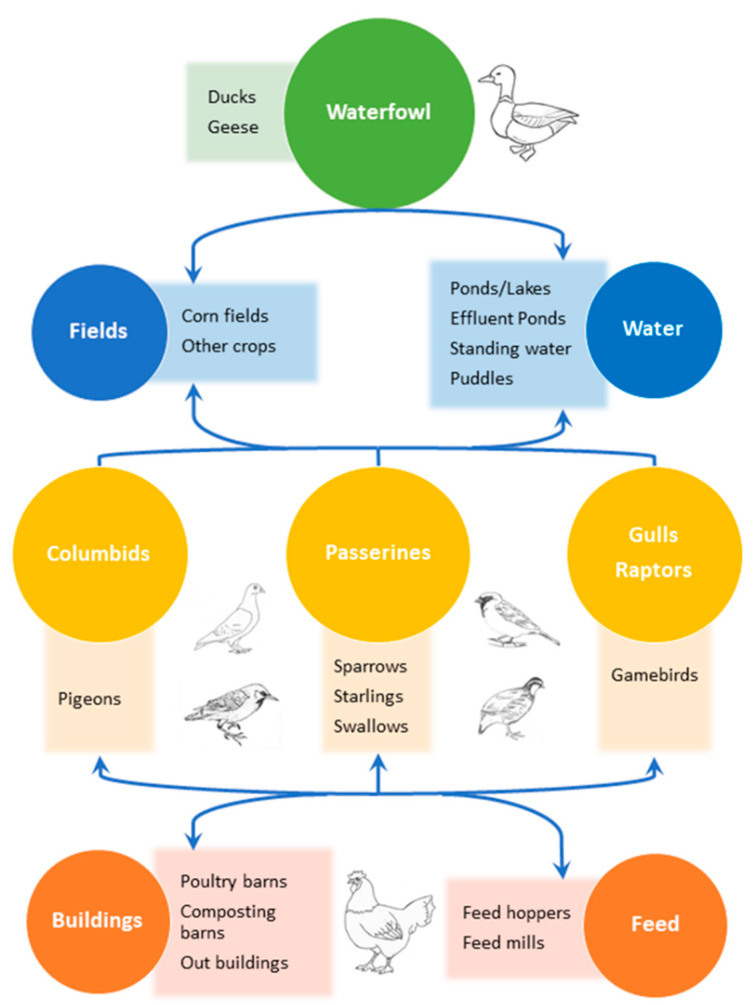
Potential risk paths for transmission, carriage, or transport of avian influenza viruses from reservoir hosts to poultry via bridge species.

A useful concept for characterizing the potential role synanthropic species might play in IAV dynamics at the wildlife–agricultural interface is the idea of bridge hosts [1,9]. Bridge hosts can be defined as non-maintenance host species capable of transmitting a pathogen from a reservoir population to a target population. Maintenance hosts for IAVs are water birds (ducks, geese, shorebirds), the target population is poultry (chickens, turkeys, gamebirds), and bridge hosts are species commonly observed both in poultry facilities and nearby habitats for maintenance hosts (Figure 1). For example, bridge hosts might share water (riparian habitat, ponds, drainages) or foraging resources (e.g., crop fields) with maintenance hosts, and water (swales, puddles, canals) or foraging (spilled feed, carcasses) resources on farms. IAVs could be transmitted between host types either by direct contact or indirect contact by mechanical spread or resource contamination. Synanthropic species are likely to occupy habitats shared by both maintenance and target hosts, so evaluating what is known about IAVs in these species sheds light on the risks they may pose as bridge hosts. In this review, we systematically review what is known about IAV exposure and infection dynamics for synanthropic species in each of the relevant order of birds.

## 2. Scavengers and Raptors: Orders Accipitriformes, Cathartiformes, Strigiformes, and Falconiformes

Several bird orders include scavenger and raptor species, such as hawks, eagles, and Old World vultures (order Accipitriformes, family Accipitridae), New World vultures (order Cathartiformes, family Cathartidae), owls (order Strigiformes, families Tytonidae and Strigidae), and falcons (order Falconiformes, Falconidae). The feeding ecology of scavengers and birds of prey has the potential to bring this group of birds into contact with IAV-infected prey or carcasses. As such, IAV infections and exposures have been commonly detected in a broad diversity of hawks, eagles, owls, and vultures, e.g., [10,11,12,13,14,15,16,17,18,19,20]. Farms are frequently used by a number of species in these groups for foraging opportunities, and these birds are regularly observed at poultry facilities, including foraging on carcasses ([21,22], personal observations).

While IAV detections in the wild have been observed in a variety of raptor and vulture species, only falcons have been the subjects of experimental studies of IAV infection dynamics. Two studies that experimentally evaluated different strains of HP H5N1 in American kestrels (*Falco sparverius*) found that the birds were highly susceptible to both strains with high mortality [23,24]. Similarly, two experimental inoculations have been conducted in Gyr–Saker hybrid falcons (*F. rusticolus* × *F. cherrua*) [25,26]. Both studies found the birds were highly susceptible to IAVs with high mortality for HP viruses. Interestingly, one study confirmed IAV transmission from the ingestion of infected chickens [26]. While these studies suggest that IAV exposure and infections are not uncommon in vultures and raptors and that some of these species are regular visitors to poultry farms, these birds may not be likely to play a role as bridge hosts in the trafficking of IAVs to poultry. Most birds of prey are relatively solitary and are therefore less likely to transmit viruses to conspecifics if they are infected. In addition, if an infected raptor or vulture did encounter poultry, the interaction would likely involve consuming poultry as prey or foraging on a carcass. In either case, onward transmission is unlikely.

## 3. Cattle Egrets, Herons, Bitterns: Order Pelecaniformes, Family Aredeidae

The order Pelecaniformes includes waterbirds in the family Aredeidae, which is comprised of egrets, herons and bitterns. While several species from this family might occur on farms, especially small-scale farms with nearby wetlands, the cattle egret (*Bubulcus ibis*) is the most likely to exhibit synanthropic tendencies. Ibises (family Threskiornithidae) are another possibility. While ibises were historically associated almost exclusively with wetlands, in areas where available wetland habitat has declined, some ibis species have begun colonizing human modified landscapes.

Cattle egrets were historically distributed primarily in Africa, but this species has greatly expanded its range due to land clearing, such that cattle egrets are now found across the globe [27]. While some populations are migratory, many are year-round residents. A study of wild birds at the wildlife–agricultural interface in Mexico found that cattle egrets were common in both wetland and farm habitats with individuals observed in poultry stalls, implicating this species as a potential bridge host. Further, an epidemiological study of risk factors associated with IAV outbreaks in ostrich farms in South Africa found that farms that reported the presence of African sacred ibis (*Threskiornis aethiopicus*) and Hadeda ibis (*Bostrychia hagedash*) were significantly more likely to have experienced an outbreak [28]. 

Field studies of IAV exposures in cattle egrets have been relatively few. A wide-ranging study of wild birds in California in the United States (US) collected cloacal swabs from 14 cattle egrets, two of which (14%) were positive for IAV viral RNA by rRT-PCR [29]. Another study in Zimbabwe found that while cattle egrets were a dominant species occurring in wetlands, villages, and on farms, none of the 166 fecal swabs were positive for IAV viral RNA [1]. Nonetheless, these authors suggest cattle egrets may be a likely IAV bridge host due to their distribution in both wetland and farm habitats. Further support for that idea comes from a field survey in Egypt [30]. In that study, an HP H5 virus was isolated from one of sixty cattle egrets sampled near a broiler farm infected with the same virus. Similarly, a field study of fecal samples from cattle egrets at a landfill in Spain isolated an H16N3 virus from 1/116 samples. While these studies suggest at least a low-level prevalence of IAVs in cattle egrets in the wild, we only found one experimental study that evaluated infection dynamics. In that study [31], six cattle egrets were experimentally inoculated with an HP H5N1 virus. While all individuals were highly susceptible and either died or were euthanized, only a low level of viral RNA shedding was observed, and none of the five contact chickens became infected. 

Several species of ibis have been studied as potential hosts of IAVs in different regions of the world. In an observational survey of birds in poultry facilities, Australian white ibises (*Threskiornis molucca*) were commonly observed foraging in poultry facilities, including direct contact with free-ranging chickens [32]. The same study showed a 24% seroprevalence in these birds from 180 samples collected in three different years. Two studies report on IAVs in African sacred ibis. A study of French wetlands where the birds are considered invasive found high seroprevalence, with 64% of 111 birds positive for antibodies reactive to H5 [33]. In South Africa, a molecular epidemiological survey to identify the route of introduction of H7 IAVs into ostrich farms implicated African sacred ibis in an introduction to ostriches based on phylogenetic evidence [34]. In another year, analysis suggested that the sacred ibis became infected after exposure to infected ostriches. Thus, these authors suggest sacred ibises may act as bridge hosts for ostrich farms. Of importance, this work suggests sacred ibises can move IAVs to and from poultry facilities.

In an observational study of birds that used both wetland and agricultural habitats in Mexico, white-faced ibis (*Plegadis chii*) were among the most commonly identified species that used multiple habitats, and these birds were also observed in barn interiors [35]. While no information is available on infection or exposure to IAVs in this species, it may be an important species for further study given its synanthropic habits. In the US, white ibises (*Eudocimus albus*) have become increasingly common in human modified landscapes. A serosurvey of white ibis in South Florida in the US found that more than 70% of the birds were seropositive for a broad spectrum of HA subtypes [36]. H6s were the most common, with significant exposures to H1, H9, H11, and H12. H2, H7, and H10 were also represented, but exposures to H3, H4, and H8 were uncommon. In experimental inoculations of white ibis reported in the same study, all birds inoculated with an H6N1 or H11N9 became infected and seroconverted, including contact birds. Viral RNA levels were much higher for cloacal swabs compared to oral swabs for both subtypes. Consistent with the results of the serosurvey, no birds became infected after inoculation with an H3N8 virus.

## 4. Gulls: Order Charadriiformes, Family Laridae

Gulls are in the order Charadriiformes in the family Laridae. These birds are considered major reservoir hosts of avian IAVs [2,37], especially of H13 and H16 viruses [38]. Large-scale surveillance of gulls has shown that infection prevalence can top 50% in many gull populations [38,39,40]. However, the H13 and H16 subtypes that make up a majority of infections in gulls are rarely isolated in poultry, and experimental inoculation of chickens and turkeys with H13 gull viruses showed that poultry are generally resistant to infection [41]. However, other virus subtypes are sometimes isolated from gulls, and many gull species exhibit synanthropic behavior, so these species might play a role in the spillover of IAVs of consequence to poultry. Black-headed gulls (*Chroicocephalus ridibundus*) were commonly observed on a poultry farm in Switzerland [21], and black-headed gulls and lesser black backed gulls (*Larus fuscus*) were frequent visitors to poultry farms in the Netherlands [22]. Additionally, an epidemiological assessment of risk factors associated with HP IAV outbreaks on ostrich farms in South Africa showed that the presence of gulls was associated with an increased likelihood of an outbreak [28].

In an extensive survey of laughing gulls (*Leucophaeus atricilla*) at Delaware Bay in the US, microneutralization tests showed the birds were exposed to a variety of HA subtypes, including H1, H5, H6, H9, and H11 [42]. In addition, many gull species appear to be susceptible to HP IAVs. For example, an HP H5N1 virus was isolated from a black-headed gull in Hong Kong [43] and HP H5N8 viruses were detected in six species of gulls in Germany during a widespread outbreak in 2016 [19]. On the other hand, ring-billed gulls (*Larus delawarensis*) experimentally infected with HP H5N2 collected from a poultry outbreak in Pennsylvania in the US did not exhibit clinical signs, and viral shedding was limited and only evident for 1–2 days [44]. During IAV surveillance sampling in Chile, a LP H5N9 virus was isolated from a kelp gull (*Larus dominicanus*), a species with synanthropic tendencies that can be found in urban areas, on farms, and as a part of large, mixed flocks [45]. Overall, while gulls are primarily associated with the circulation of H13 and H16 viruses, these birds are clearly capable of shedding viruses relevant to poultry. 

## 5. Pheasants, Turkeys, Peafowl, Old World Quail, New World Quail: Order Galliformes

The primary species of interest within the order Galliformes that exhibit a potential for synanthropic behavior are pheasants, turkeys, peafowl, and quail within the families Phasianidae and Odontophoridae. Many of the species in these families have been domesticated (e.g., turkeys, pheasants, and quail), and are frequently raised on gamebird and backyard farms and found at live bird markets. As such, many studies have documented that domesticated individuals of birds in these families are susceptible to and shed avian IAVs. However, less is known about IAV dynamics in wild individuals or the frequency of contacts between wild and domestic individuals. Wild individuals of domesticated species pose a potential risk for IAV transmission because farms can act as attractants to wild individuals in search of food resources or drawn by the presence of similar individuals.

A serosurvey of wild pheasants (*Phasianus colchicus*) in Italy (1995–2002) found an overall seroprevalence of 12.3% across 219 samples (yearly range 0.0%–42.5%), including two seroconversions in recaptured birds at subsequent time points [46]. No antibodies to H5 or H7 IAVs were detected. A similar study of wild bobwhite quail (*Colinus virginianus*) in Texas, US from 2009–2011 tested 652 tracheal swabs, 668 cloacal swabs, and 44 serum samples from wild-captured and hunter-harvested bobwhite quail [47]. No virus was isolated and no antibodies reactive to IAV were detected, but 13 cloacal swabs and 5 tracheal swabs were positive for viral RNA by rRT-PCR, with another 100 swabs showing suspect positive results (Ct > 35). The authors conclude that IAVs are only present at low prevalence in wild bobwhite populations.

Domestic turkeys are considered one of the most permissive domesticated hosts of IAVs, which suggests that wild turkeys (*Meleagris gallopavo*) could be important wildlife hosts for IAVs if they are similarly susceptible [48]. While we did not find any studies that experimentally assessed wild turkey susceptibility to IAVs, field surveillance of wild turkeys indicates the species is not regularly exposed to IAVs. We evaluated nine studies that collectively included 1173 serum samples from across the US and 864 swab samples from the US and Canada (Table 1) [49,50,51,52,53,54,55,56,57]. Only a single weak positive sample was detected by agar gel immunodiffusion (AGID) from a live wild turkey sampled in California, US [52]. Consequently, while wild turkeys do not appear to play a role in the natural circulation of IAVs, if they are susceptible, they might play a role in IAV dynamics as a spillover species during IAV outbreak in poultry [48]. One study explored this possibility with a field survey of hunter-harvested wild turkeys from Minnesota counties in the US that had experienced recent HP H5N2 poultry infections [55]. They did not detect any infections from swab samples, which suggests wild turkeys were not commonly infected; however, an alternative explanation is that, because the outbreak virus was lethal in domestic turkeys, exposed wild turkeys may have suffered mortality and were not available for sampling. Thus, while field data do not support the idea that wild turkeys are likely to significantly contribute to IAV maintenance or as a bridge host, experimental infection studies could provide risk information by confirming whether this species is permissive to infection.

## 6. Pigeons, Doves: Order Columbiformes, Family Columbidae

Rock doves (*Columba livia*), commonly referred to as pigeons, are members of the family Columbidae in the order Columbiformes. Pigeons are widely known for their synanthropic behavior and are frequently associated with poultry facilities. Consequently, pigeons have been the frequent subject of IAV research, including a comprehensive review published in 2016 [58]. Overall, the review highlights that pigeons can become infected with IAVs, but that viral shedding is generally very limited and only occurs for brief periods. The author suggests that if pigeons do play a role in IAV transmission, mechanical or fomite transmission is the most likely route of transmission, possibly by contaminated feet or feathers [58]. We refer readers to the review for details on the studies leading to these overall conclusions.

Many studies have been published since the review, and we highlight a few. An experimental inoculation study, testing both LP H5N8 isolated from a poultry outbreak and a wild bird H4N6, corroborated limited shedding in infected birds, but highlighted that some individuals shed potentially infectious doses [59]. Limited shedding reduces the probability of onward transmission, and most studies show limited or no transmission to naïve contacts. The emergence of an avian H7N9 virus in China in 2013 resulted in evaluations of that virus in pigeons after the virus was isolated from both wild and domestic pigeons [60]. Pigeons experimentally inoculated with the emergent H7N9 virus showed high survival and minimal shedding of the virus [60], a finding similar to those found in two other studies [61,62]. Another experimental study of the emergent H7N9 virus simulated a live bird market where pigeons were exposed to infected animals in cages stacked above them; none of the pigeons became infected [63]. A study of pigeons in Egypt naturally infected with an HP H5N1 virus corroborated that pigeons do become infected with these viruses [64]. Similar to most studies of IAV in pigeons, a test of another recently-emerged virus, HP H5N8 clade 2.3.4.4, showed that most experimentally inoculated pigeons became infected, but with limited viral shedding across a brief period and no transmission to contact pigeons [65]. In another study using the HP H5N8 clade 2.3.4.4 virus, no inoculated pigeons showed clinical signs or shed virus [66]. Another experimental inoculation with an HP clade 2.3.4.4 virus, but in this case an H5N6 virus, showed that several pigeons became infected and shed virus at moderately high levels; nonetheless, no contact chickens became infected [67]. In total, relatively recent studies of IAVs in pigeons have characterized the infection dynamics for newly-emerged viruses, but overall the general patterns seen across many prior studies confirm that pigeons can generally become infected with a variety of IAVs, usually shed limited viral quantities for brief periods of time and rarely transmit to naïve contacts, but some individuals and some IAV strains can lead to productive infections and transmission, so pigeons cannot be completely ruled out as a potential risk for spillover into poultry.

## 7. Passeriformes: Thrushes, Finches, Swallows, Starlings, Sparrows

The order Passeriformes is the most speciose among the bird orders, including more than half of all known bird species divided into nearly 150 bird families. Birds in this order are commonly known as passerines, songbirds, or perching birds. While there are many thousands of passerine species, most synanthropic species are restricted to seven families: Corvidae (crows, ravens, jays, and magpies), Hirundae (swallows), Sturnidae (starlings), Turdidae (thrushes and robins), Passeridae (Old World sparrows), Fringillidae (finches), and Icteridae (blackbirds and grackles). Passerines from other families might occur or even be common on farms, but most species outside of these families are not likely to play a significant role in IAV dynamics based on behavior (i.e., limited interaction with poultry or shared resources) or physiological characteristics. For example, many passerines are quite small, and would have to shed significant virus titers to introduce adequate levels of virus for transmission, especially for non-flocking birds. Furthermore, many passerines are primarily insectivores, so they would not be expected to share and contaminate food resources with potential bridge or poultry species.

An abundant species in sub-Saharan Africa, the red-billed quelea (*Quelea quelea*) in the family Ploceidae, is considered a serious pest of cereal crops. We did not identify other birds in this family that express synanthropic tendencies. Quelea are commonly attracted to poultry feed and are therefore common visitors to poultry farms. In a study of the wildlife–agricultural interface in Zimbabwe, these birds were classified as a likely bridge host due to their regular presence on farms and IAV viral RNA detections [1].

### 7.1. Corvidae: Crows, Ravens, Jays and Magpies

Corvids are widespread throughout the globe. Most species are omnivorous generalist feeders that frequently forage opportunistically [68]. Many synanthropic corvids are avid scavengers and are therefore attracted to farms where carcasses might be available. No single species is common throughout the globe, but many species of crows, ravens (*Corvus* spp.) and magpies (*Pica* spp.) are often associated with farms, with several studies documenting their consistent presence in Canada, Germany and the Netherlands [21,69,70]. In Germany, carrion crows (*C. corone*) and ravens (*C. corax*) were both commonly detected on farms [21], and in the Netherlands magpies (*Pica pica*), carrion crows and jackdaws (*C. monedula*) were all observed on a poultry farm, with specific observations of these species overlapping with free-ranging poultry and foraging on eggs [22]. An epidemiologic investigation of risk factors for predicting HP H5N1 outbreaks on farms in Bangladesh identified the presence of dead house crows (*C. splendens*), a common synanthropic resident throughout Bangladesh known for its scavenging behavior, on or near farms as the most predictive risk factor associated with IAV outbreaks [71].

While we did not find any studies reporting antibody detections or experimental inoculations of corvids, IAVs have been isolated from several corvid species in several countries, especially from dead crows found near HP H5N1 outbreak sites. In 2004 in Japan, HP H5N1 was isolated from nine jungle crows (*C. macrorhynchos*) after an outbreak in poultry [72,73]. The birds were all found within a 30 km radius of an outbreak farm where the species was commonly observed, suggesting that they likely became infected at the outbreak facility. Similarly, HP H5N1 was isolated from two jungle crows in India after poultry outbreaks in that country [74], and from carrion and jungle crows near outbreak sites in Bangladesh [75]. More recently, HP H5N8 viruses in the 2.3.4.4 clade were isolated from carrion crows and magpies after outbreaks throughout the country [76]. In contrast, a large-scale study of hooded crows in Italy that assessed both exposure and incidence did not find any evidence of IAVs in this species [77]. These studies suggest that corvids are generally susceptible to HP IAVs, but limited information is available on LP viruses in these birds or infection dynamics. Moreover, all of these isolations indicate that corvids were infected after outbreaks occurred, so more information is needed to determine whether transmission from corvids to target species presents a risk to poultry.

### 7.2. Hirundae: Swallows

Swallows are commonly found in open habitats, and as such they can be very common on some farms. These species are almost exclusively insectivores, which may limit their interaction with poultry or shared resources. However, given their occupancy and abundance at many poultry facilities where they commonly use farm structures for nesting, these birds are a priority for research to understand their potential role in IAV dynamics [1,35,69,78]. Specifically, barn swallows (*Hirundo rustica*) are geographically widespread, and several studies have documented the presence of this species on poultry farms. Barn swallows were found to be very common on poultry facilities in Canada and were observed entering barns [69]. A similar study focused on identifying potential IAV bridge hosts in Zimbabwe also identified barn swallows as a species of concern [1]. In that study and a follow-up investigation, not only were barn swallows common on farms, but sampling showed the presence of IAV viral RNA in swabs from some individuals [79]. Barn swallows were also identified as potential hosts for IAV transmission in a study of the wildlife–agricultural interface in Mexico based on abundance on farms and wetland habitats, combined with a detection of an H7N3 IAV during an outbreak [35]. Furthermore, swallows were one of the most commonly captured birds in a study of potential bridge hosts at outbreak farms in Iowa in the US [78].

Surveillance studies confirm swallows can be infected with IAVs. In addition, to the study above, H4, H9, H10, and H11 viruses were isolated from three swallows sampled in Slovakia [80]. In Vietnam, an HP H5N1 was isolated from an apparently healthy barn swallow [81]. While these isolations indicate that swallows can be infected with IAVs, we did not find any experimental studies that characterized infection dynamics. Moreover, given the insectivorous nature of these birds, further study is needed to understand if these birds are infected from occasional spillovers from poultry or from interactions with IAV maintenance hosts.

### 7.3. Sturnidae: Starlings

European starlings (*Sturnus vulgaris*) are the main synanthropic species in the Sturnidae family. Starlings were introduced into North America in the late 1800s, and that introduction may be the most successful introduction of all time, with starlings common throughout the continent except at northern latitudes. Starlings have been successfully introduced into several other regions, and the species is now common in North America, Eurasia, and parts of South America and Australia. Starlings have a broad diet which allows them to adapt opportunistically to local resources. They are often attracted to livestock feed and have therefore become common occupants on farms. Starlings are cavity nesters, which poses a risk to poultry facilities when starlings enter barns, breaches, or other farm structures in search of nesting substrate. 

Starlings were only occasionally observed on poultry farms in Germany [21], and were moderately common on a poultry facility in the late summer in the Netherlands, where they were often observed foraging [22]. In contrast, starlings were one of the most abundant birds observed on poultry farms in Canada and were identified as a high priority species for further study to assess IAV spillover risk [69]. In Canada, starlings were not only abundant, but were sometimes observed in flocks of more than a thousand birds, were observed entering barns through roof vents and holes near eaves, and were also observed using nearby croplands and wetlands.

Starlings have been sampled for IAV exposure and infections in a comparatively large number of studies over time. We reviewed 14 studies (Table 2). Across the studies, seroprevalence was relatively low, with only six birds identified with antibodies reactive to IAV out of 1032 birds sampled (0.58%). IAV or viral RNA was detected in 26 birds across 1451 birds sampled (1.79%). The lower seroprevalence rate compared to prevalence suggests detectable antibodies might be relatively transient in this species.

Experimental studies of starlings have also been relatively common. We identified seven experimental studies that evaluated seven different IAV subtypes. In the earliest study that we found, starlings were inoculated with an HP H7N7 IAV [82]. All birds replicated significant levels of virus in multiple tissues and all birds died, including naïve contacts. Two studies evaluated HP H5N1 viruses in starlings. In the first, no disease, mortality, lesions, or viral antigens were detected in tissues [83]. In the second study, four different strains of HP H5N1 were evaluated [84]. Most birds shed high levels of virus in oral swabs, but only one contact became infected and no birds died. The authors suggest starlings might act as an IAV spillover host, but also suggest that limited contact transmission would likely prevent sustained transmission. 

A study of a LP H3N8 virus in starlings showed that most birds shed viral RNA orally with rare cloacal shedding, but there was no contact transmission [85]. Another experimental inoculation of LP viruses, this time an H2N3 from chickens and an H4N2 from waterfowl, showed that all inoculated starlings became infected, shed moderate amounts of viral RNA, and seroconverted, but shedding was higher for cloacal swabs compared to oral swabs [86]. A study that evaluated the emergent H7N9 virus compared birds inoculated with low, medium, and high doses of virus [87]. Only half the birds in the high dose group became infected, with high levels of viral RNA detected from oral swabs. In a study of three strains of clade 2.3.4.4 H5N8 and H5N2 IAVs, no birds showed clinical signs or evidence of infection, but all birds seroconverted [88]. Overall, these studies suggest that IAVs regularly spill over into starlings, but inconsistent viral shedding and limited contact transmission indicate that the role starlings might play in IAV epidemiology is likely strain-dependent.

### 7.4. Turdidae: Thrushes

Most thrush species do not exhibit synanthropic tendencies, but in North America the American robin (*Turdus migratorius*) is a very common species found throughout urban and rural habitats. American robins are commonly found on poultry farms [69,78], especially during the breeding season when they frequently use farm structures for nesting substrate. American robins primarily forage on insects and small fruits, so they are not generally attracted to poultry feed, eggs or carcasses. Nonetheless, their consistent presence on poultry farms indicates this species could pose a risk as an IAV bridge host. In Europe, the Eurasian blackbird (*T. merula*) may play a similar role, as it was commonly observed on poultry farms in both Germany and the Netherlands [21,22]. 

In a wildlife epidemiologic investigation in Iowa in the US, American robins were one of the most commonly captured species on outbreak premises [78]. While no infections were detected in the robins, two birds were seropositive for antibodies reactive to the clade 2.3.4.4 HP H5N8 outbreak virus. In a very large-scale surveillance study of IAVs in wild birds in the US, IAV viral RNA was detected in both American robins and Swainson’s Thrushes (*Catharus ustulatus*), with 5/133 and 10/265 cloacal samples positive for viral RNA, respectively [100].

An experimental inoculation study of American robins using three different strains of the 2.3.4.4 clade HP H5 viruses showed that robins were highly susceptible to the viruses (22/25 robins were infected), with high levels of viral shedding detected in oral swabs [101]. Given the ubiquity of American robins on farms in North America and potential susceptibility to IAVs, this species should be a high priority for further study to assess the risk these birds may play in potential spillover transmission to poultry. A thrush species found in Japan that is similar to the American robin is the pale thrush (*T*. *pallidus*). This species is known to occupy edge habitats, and was therefore studied as a potential bridge host for IAVs [102]. In that study, the birds were inoculated with an HP H5N1 virus. The thrushes were highly susceptible to infection, and infected birds had high virus titers in lung tissues. Collectively, these studies suggest that thrushes may be susceptible to multiple IAVs, but more work is needed to assess the risk of spillover associated with this family of birds.

### 7.5. Passeridae: Old World Sparrows

Two species within the Passeridae family are synanthropes, the Eurasian tree sparrow (*Passer montanus*) and the house sparrow (*Passer domesticus*). Both species are very common in human-modified landscapes along the urban to rural divide. House sparrows were introduced to the Americas, but are now widespread. In surveys of birds at poultry facilities, house sparrows were very common on farms in Germany, the Netherlands, Canada, southeastern Brazil and Mexico [21,22,35,69,103], where they were commonly observed in barns as well as using nearby cropland and wetlands. Many studies have sampled sparrows for IAV exposure. Across 13 studies reporting surveillance in sparrows, seroprevalence was high, at 11.4%, with much more muted prevalence at 0.64% (Table 3).

We identified a dozen experimental infection studies for sparrows. Overall, these studies generally show that Eurasian tree sparrows and house sparrows are susceptible to most studied IAVs, often shed high levels of virus, and can transmit IAV to naïve contacts. In a study of HP H7N7 in house sparrows, experimentally inoculated birds shed high levels of virus in multiple tissues and one-third of birds died, but no naïve contacts were infected [82]. House sparrows have also been challenged with HP H5N1 in three studies. In one study, house sparrows showed mild clinical signs, no lesions, no mortality, and only limited evidence of antigen in tissues [83]. Interestingly, this study found limited morbidity and mortality in starlings for this strain of HP H5N1. In another study that evaluated four HP H5N1 strains, house sparrows were susceptible to all four viral strains, shed moderate levels of virus, and experienced high mortality rates [84]. The third study of HP H5N1 in house sparrows showed that birds were highly susceptible to the virus, even at low infectious doses [104]. Similarly, one study of HP H5N1 in tree sparrows showed that most birds died of infection [105], and a second study found that all birds were infected, shed moderate levels of virus, and two of eight naïve contacts became infected [106].

Three studies of HP H5N1 in tree sparrows also studied intraspecific and interspecific transmission. In one, directly inoculated sparrows were highly susceptible to the virus, with high viral loads and mortality, but no contact tree sparrows became infected [113]. However, contact transmission did occur to chickens, and more than one-half of sparrows exposed to infected ducks showed high levels of viral RNA on swabs taken from feathers. In another study of HP H5N1 in tree sparrows, directly inoculated birds were infected and died at both low and high doses, and contact chickens became infected by water contaminated by infected sparrows [114]. A third study of H5N1 in house sparrows confirmed high susceptibility and shedding in directly inoculated birds, some contact transmission to sparrows exposed to infected chickens, and no contact transmission from chickens exposed to infected sparrows [115].

Several experimental studies have also evaluated LP IAVs in sparrows. House sparrows experimentally inoculated with a LP H3N8 showed moderate susceptibility, with muted and transient shedding and no spread to naïve contacts [85]. An experimental study of house sparrows inoculated with H9N2 showed efficient transmission between house sparrows and chickens in both directions [116], but birds infected with H7N9 in another study showed limited contact transmission, even though infected birds shed high titers of virus [117]. In summary, house sparrows appear to be susceptible to most strains of HP IAVs, with moderate to high shedding and contact transmission, but their response to LP IAVs may be more variable. 

### 7.6. Fringillidae: Finches

Fringillidae, the family of finches, is comprised of a broad spectrum of species, and only a few of those species exhibit synanthropic tendencies, most notably the house finch (*Haemorhous mexicanus*) in North America and the common chaffinch (*Fringilla coelebs*) in Eurasia. In North America, house finches were frequent visitors to both poultry farms and wetland areas [69], and in Germany common chaffinches were commonly found within 500 m of poultry enclosures, with some observations made of chaffinches within enclosures [21]. While these birds are relatively common visitors to farms, relatively little research has evaluated IAVs in these species. In a surveillance study of migratory birds in Helgoland in the North Sea (Germany), samples from 131 chaffinches did not result in any IAV isolations [118]. In California in the US, samples from 420 house finches resulted in only two samples positive for viral RNA. These results suggest finches are unlikely to play a role in IAV epidemiology, but more work is needed to confirm these results.

### 7.7. Icteridae: Blackbirds and Grackles

Icterids are another speciose family within the passerines. A handful of species within this family, such as grackles (*Quiscalus* spp.), cowbirds (*Molothrus* spp.) and blackbirds (*Agelaius* spp. and *Euphagus* spp.), are synanthropic, and are commonly found on farms. Interestingly, red-winged blackbirds (*A. phoeniceus*) are common on farms and nest in wetlands. Observations on farms in Canada found that icterids were commonly observed with Brewers blackbirds *(E. cyanocephalus*), red-winged blackbirds, common grackles (*Q. quiscula*) and brown-headed cowbirds (*M. ater*), all observed visiting farms [69]. Common grackles were one of the most common birds on the farms, and were flagged as a high priority species for further research, based on their use of wetlands and crops and their observed presence in the immediate barn areas of farms. A similar study in Mexico identified great-tailed grackles (*Q. mexicanus*) as potential bridge hosts, based on their common presence on farms, observations of the birds in poultry stalls, and a prior detection of an H7N3 IAV during an outbreak of that virus [35]. In an experimental inoculation with LP H5N2 and H7N2 viruses, directly inoculated red-winged blackbirds were infected and shed significant amounts of virus [119]. However, in a simulated barnyard experiment where blackbirds shared a room with infected mallards, none of the birds became infected through contact with infected individuals or contaminated resources. These studies suggest that icterids may play a role in IAV spillover, but more research is needed to determine the generality of these findings.

## 8. Conclusions and Future Directions

As this review has detailed, a diversity of avian species display synanthropic behaviors, and have the potential to bridge IAVs from maintenance hosts to poultry. While most species reviewed here are not regularly exposed to IAVs, most avian species are susceptible to infection, and shed moderate levels of virus for at least some IAV strains. Even in the case of pigeons that have been shown to infrequently shed significant levels of virus and are considered to play a non-significant role in IAV dynamics [58], some IAV strains can replicate well in some individuals. Consequently, an overarching strategy to minimize the risk of IAV introductions to poultry by synanthropic species is to maintain rigorous biosafety practices that minimize the attractiveness of farm resources to these species.

**Figure 2:**
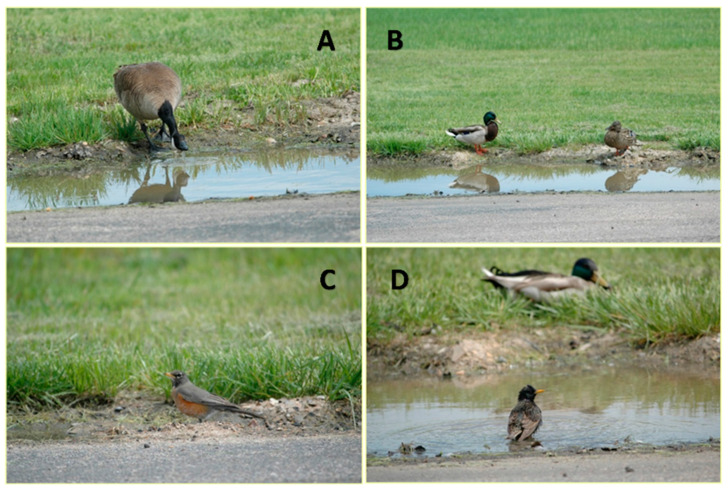
(**A**) Canada goose, (**B**) mallard, (**C**) American robin, (**D**) European starling (mallard in background) all sharing the same puddle over a short period of time.

Understanding how synanthropic species might traffic IAVs onto poultry facilities suggests the need for biosecurity practices to reduce risk from these birds. A high priority is to reduce opportunities for direct contact with poultry by reducing the possibility of incursions into buildings when wild birds can enter barns through open eaves, vents, building breaches, or insecure doorways. Similarly, biosecurity practices aimed at reducing opportunities for indirect transmission via contaminated resources are also a high priority. Spilled feed, carcasses, eggs, and other potential food resources attractive to wild birds should be minimized by consistent and frequent maintenance of feed machinery to identify spills. Carcasses and eggs should always be inaccessible to wildlife. Frequent perimeter and ground checks to identify and remove feed, carcasses, or eggs can be used to reduce wild bird attraction to farm premises [22]. Another potential attractant with a high probability of IAV contamination is puddles or flooded swales on farms. Many farms have low-lying areas that can become flooded and harbor persistent puddles. Because all bird species need water for drinking and bathing, puddles can act as an attractant and a common gathering area, and increase the probability of interspecies interactions and the sharing of IAVs (Figure 2). Thus, minimizing the development of puddles by leveling ground could reduce contact between individuals and species, thereby reducing the risk of sharing IAVs through contaminated water [78].

The available information for some synanthropic species is relatively sparse, so the continued study of these species will aid in understanding the risk they may pose in IAV spillover to poultry. The combination of broadly diverse IAV strains, surveillance methodologies, and experimental approaches all limit general inference across studies and species. For example, many of the surveillance studies reviewed only evaluated exposure or incidence for H5 and/or H7 virus subtypes, thus limiting inference across all IAVs. Similarly, while IAV excretion can vary across host species and viral strains, many surveillance studies only collected oral, cloacal or fecal swabs, and may therefore have missed infections when excretion is primarily associated with a different route than that sampled. Surveillance studies evaluating multiple routes of viral excretion and IAV strain-independent testing provide the basis for broad inference. When such studies are not available or feasible, multiple studies may be needed to broadly evaluate the role a given host may play in IAV spillover.

As new IAVs emerge, surveillance and experimental inoculation studies can be used to assess whether synanthropes pose a risk for high-consequence strains. Moreover, studies that go beyond experimental inoculations and address transmission pathways would provide useful information on whether demonstrated susceptibility and replication translate to transmission. In particular, studying the role of behavior may provide important insights into the risks a particular species poses for potentially bridging IAVs to poultry. For example, while American robins may be common on poultry facilities and readily excrete IAVs, these birds primarily use farms for nesting substrate. Their feeding ecology limits their use of spilled feed or other resources, thus reducing the likelihood they would contaminate shared resources other than water. In contrast, house sparrows and European starlings are attracted to the same food resources as poultry, thus increasing the risk of these species coming into direct contact with poultry.

Another high priority for future research is more studies that characterize farm use by wild birds. While several studies have been published [1,21,22,45,69], these studies require significant effort, so data are often limited to a handful of farms which limits inference. In addition, information for some geographic regions is scant. More information would increase our ability to identify and rank high-priority species, and to begin to understand regional differences.

## Figures and Tables

**Table 1:** Avian influenza A virus surveillance of wild turkeys in the US and Canada.

Location	Sampling Year(s)	Serosurvey (N Sampled)	Serosurvey (N Positive)	Virus/RNA Detection (N Sampled)	Virus/RNA Detection (N Positive)	Citation
PA, US *	1983–1984	7	0	--	--	[50]
VA, US *	1983–1984	--	--	62	0	[50]
TX, US	1983–1985	440	0	511	0	[42]
USA	1981–1986	210	0	--	--	[43]
AR, US	1986	44	0	--	--	[44]
CA, US	1986–1996	383	1	--	--	[45]
TX, US	2001	70	0	--	--	[46]
GA, FL, US	2005–2008	19	0	--	--	[47]
MN, US	2015	--	--	84	0	[48]
Ontario, Canada	2011–13, 2015	--	--	207	0	[49]
Totals		1173	1 (0.09%)	864	0	

* Samples collected from the quarantine zone of outbreak premises.

**Table 2:** Avian influenza A virus surveillance of European starlings.

Location	Sampling Year(s)	Serosurvey (N Sampled)	Serosurvey (N Positive)	Virus/RNA Detection (N Sampled)	Virus/RNA Detection (N Positive)	Citation
Israel	1978–1979	--	--	42	1 H1	[89]
Great Britain	1981	--	--	?	1 H7	[90]
Israel	1981	--	--	282	1	[91,92]
Australia	1985	--	--	<208	1 H7N7	[82]
Ohio US	1988?	868	0	--	--	[93]
Georgia US	1999	15	0	--	--	[94]
Slovenia	2004	--	--	670	1	[95]
Russia	2007	--	--	5	1	[96]
Iraq	2007	60	0	--	--	[97]
Ohio US	2007–2008	--	--	328	21	[86]
Australia	2008–2009	--	--	50	0	[98]
Iowa US	2015	69	6	69	1	[78]
Iowa US	2015–2016	5	0	5	0	[99]
Totals		1032	6 (0.58%)	1451	26 (1.79%)	

**Table 3:** Avian influenza A virus surveillance of house sparrows and tree sparrows.

Location and Species	Sampling Year(s)	Serosurvey (N Sampled)	Serosurvey (N Positive)	Virus/RNA Detection (N Sampled)	Virus/RNA Detection (N Positive)	Citation
Australia	1985	?	1 H7N7	--	--	[82]
Hong Kong, tree sparrow	2002	--	--	1	1 H5N1	[43]
China, tree sparrow	2004	--	--	38	4	[107]
Thailand, house sparrow	2004–2008	--	--	118	0	[108]
China, tree sparrow	2008	--	--	68	1 H5N1	[106]
California US, house sparrow	2005–2008	--	--	77	1	[29]
China, tree sparrow	2011	800	94	1300	0	[105]
Indonesia, tree sparrow	2010	--	--	1	1	[109]
China, tree sparrow	2013	--	--	?	1	[110]
China, tree sparrow	2006–2009	--	--	?	4	[111]
Ohio US, house sparrow		--	--	373	0	[93]
Iowa US, house sparrow	2015–2016	44	0	44	0	[99]
Mexico, house sparrow	2010–2012	--	--	9	5	[112]
Totals		844	94 (11.14%)	2029	13 (0.64%)	
